# Real-world use of defibrotide for veno-occlusive disease/sinusoidal obstruction syndrome: the DEFIFrance Registry Study

**DOI:** 10.1038/s41409-022-01900-6

**Published:** 2022-12-23

**Authors:** Mohamad Mohty, Didier Blaise, Régis Peffault de Latour, Myriam Labopin, Jean Henri Bourhis, Benedicte Bruno, Patrice Ceballos, Marie Detrait, Virginie Gandemer, Anne Huynh, Faezeh Izadifar-Legrand, Charlotte Jubert, Hélène Labussière-Wallet, Delphine Lebon, Sébastien Maury, Catherine Paillard, Cécile Pochon, Cecile Renard, Fanny Rialland, Pascale Schneider, Anne Sirvent, Kobby Asubonteng, Gwennaëlle Guindeuil, Ibrahim Yakoub-Agha, Jean-Hugues Dalle

**Affiliations:** 1 Hôpital St Antoine, INSERM UMRs 938, Université Sorbonne, Paris, France; 2 Institut Paoli-Calmettes, Management Sport Cancer Laboratoire (MSC), Aix Marseille Université, Marseille, France; 3 Hôpital Saint-Louis, Université Paris Cité, Paris, France; 4 Département d’Hématologie, Institut Gustave Roussy, Villejuif, France; 5 CHU de Lille, Hôpital Jeanne de Flandre, Lille, France; 6 Département d’Hématologie, CHU de Montpellier, Montpellier, France; 7 CHRU de Nancy, Service d’hématologie, Hôpitaux de Brabois, Vandoeuvre-lès-Nancy, France; 8 University Hospital of Rennes, Rennes, France; 9 Département d’Hématologie, Institut Universitaire du Cancer de Toulouse—Oncopole, Toulouse, France; 10 Département d’Hémato-oncologie Pédiatrique, CHU Bordeaux, Bordeaux, France; 11 Hôpital Lyon Sud, Pierre Bénite, France; 12 CHU Amiens-Picardie, Amiens, France; 13 Hôpital Henri Mondor, Université Paris-Est Créteil Val de Marne (UPEC), Créteil, France; 14 Département d’Hémato-oncologie Pédiatrique, CHU de Hautepierre, Strasbourg, France; 15 Institut d’Hématologie et d’Oncologie Pédiatrique, Lyon, France; 16 CHU de Nantes, Hôpital Femme-Enfant-Adolescent, Nantes, France; 17 Département d’Hémato-oncologie Pédiatrique, CHU de Rouen, Rouen, France; 18 CHU de Montpellier, Hôpital A De Villeneuve, Montpellier, France; 19 Jazz Pharmaceuticals, Philadelphia, PA USA; 20 Jazz Pharmaceuticals, Lyon, France; 21 CHU de Lille, INSERM U1286, Infinite, Université de Lille, Lille, France; 22 Hôpital Robert-Debré, GHU APHP Nord et Université de Paris, Paris, France

**Keywords:** Drug therapy, Haematopoietic stem cells

## Abstract

Veno-occlusive disease/sinusoidal obstruction syndrome (VOD/SOS) is a potentially life-threatening complication of haematopoietic cell transplantation (HCT) conditioning. The DEFIFrance post-marketing registry study evaluated effectiveness and safety in patients who received defibrotide. It collected retrospective/prospective patient data from 53 French HCT centres from July 2014 to March 2020. Primary endpoints were survival and complete response (CR; total serum bilirubin <2 mg/dL, multiorgan failure resolution) at Day 100 post-HCT among patients with severe/very severe VOD/SOS. A secondary endpoint was evaluation of treatment-emergent serious adverse events (TESAEs) of interest. Of 798 patients analysed, 251 and 81 received defibrotide treatment for severe/very severe VOD/SOS and mild/moderate VOD/SOS post-HCT, respectively; 381 received defibrotide for VOD/SOS prophylaxis. In patients with severe/very severe VOD/SOS post-HCT, Kaplan–Meier–estimated CR at Day 100 was 74% (95% confidence interval [CI]: 66%, 81%). At Day 100, 137/251 (55%) were alive and in CR. Kaplan–Meier–estimated Day 100 post-HCT survival was 61% (95% CI: 55%, 67%) in patients with severe/very severe VOD/SOS. TESAEs of interest occurred in 29% of these patients; VOD/SOS-related mortality at 12 months was 15%. DEFIFrance represents the largest collection of real-world data on post-registration defibrotide use, supporting the real-world utility of defibrotide for patients with severe/very severe VOD/SOS post-HCT.

## Introduction

Veno-occlusive disease/sinusoidal obstruction syndrome (VOD/SOS) is a potentially fatal complication of haematopoietic cell transplantation (HCT). Multiorgan failure (MOF) is associated with the most severe form of VOD/SOS, and without prompt intervention, VOD/SOS with MOF may result in a mortality rate of >80% [1]]. An estimated 5% to 14% of patients who undergo HCT will develop VOD/SOS [1–3]. Individual risk of VOD/SOS depends on a number of patient risk factors (e.g. patient age, second HCT after disease relapse, HCT comorbidity index), hepatic risk factors (e.g. prior exposure to hepatotoxic or ozogamicin-containing drugs), and HCT-related risk factors (e.g. conditioning regimen, stem cell source). Myeloablative chemotherapy-based conditioning or high-dose chemotherapy in malignant haematologic disease treatment has been implicated in higher rates of VOD/SOS; however, reduced-intensity conditioning regimens do not eliminate the risk of developing VOD/SOS [1–3].

VOD/SOS results from endothelial cell activation and injury, which is typically induced by radiation or toxic metabolites from HCT conditioning regimens [4, 5]. Defibrotide acts as an endothelial cell protector and stabiliser by restoring the thrombo-fibrinolytic balance, promoting anti-inflammatory pathways, and decreasing the expression of adhesion molecules [6, 7]. Clinical studies and real-world evidence support the efficacy and safety of defibrotide for the treatment of patients with VOD/SOS. A phase 3 study in patients with VOD/SOS and MOF post-HCT demonstrated a significantly higher Day 100 post-HCT survival and complete response (CR) in patients treated with defibrotide versus historical controls [8]. Results from real-world studies of patients treated with defibrotide for severe VOD/SOS post-HCT [9, 10] or any severity of VOD/SOS post-HCT [11] have reported a Day 100 post-HCT survival of approximately 60% to 70% [9–11].

Defibrotide is approved for the treatment of hepatic VOD/SOS with renal or pulmonary dysfunction post-HCT in the United States and for severe hepatic VOD/SOS post-HCT in patients aged >1 month in the European Union, at a recommended dosage of 25 mg/kg/day for at least 21 days and until disease resolution [12, 13]. To assess the post-approval landscape of defibrotide in France, the DEFIFrance study collected real-world data on the usage, effectiveness, and safety of defibrotide from 53 French HCT centres. This analysis presents data from 381 patients who received defibrotide for prevention of VOD/SOS and 336 patients who received defibrotide for the treatment of VOD/SOS in the transplant setting.

## Methods

### Study design

This multicentre, post-marketing registry study collected retrospective and prospective real-world data on patients receiving defibrotide at 53 of 55 HCT centres in France from July 2014 to March 2020 (Supplementary Table 1); prospective data collection began in January 2017. Two centres (one paediatric and one adult) participated but did not enrol any patients. The cut-off date for this analysis (i.e. last patient last follow-up visit) was 19 March 2021.

### Eligibility criteria

Patients who received defibrotide for any reason were eligible. This included, but was not limited to, patients who received defibrotide for the treatment of VOD/SOS post-HCT or post-chemotherapy, patients who received defibrotide for VOD/SOS prophylaxis, and patients who received defibrotide for treatment of other conditions, such as transplant-associated thrombotic microangiopathy. Diagnosis of VOD/SOS was at the investigator’s discretion using standard criteria, per typical clinical practice. These criteria included, but were not limited to, hyperbilirubinaemia, hepatomegaly, ascites, and weight gain. Disease severity was graded using the European Society for Blood and Marrow Transplantation (EBMT) criteria [10, 14, 15]. VOD/SOS severity for patients aged ≥18 years was graded according to adult EBMT criteria [14]. Due to differences in the manifestations of VOD/SOS between adults and children, updated paediatric-specific EBMT criteria were published in February 2018. Patients aged <18 years were graded either retrospectively by the paediatric expert member of the Scientific Committee, if enrolled prior to publication of the updated paediatric EBMT severity grading criteria, or prospectively by the treating physician if enrolled after publication of the criteria [15]. Severity of all paediatric cases was adjudicated by the paediatric expert member of the Scientific Committee.

### Endpoints and assessments

The primary study population was patients who received defibrotide for treatment of severe/very severe VOD/SOS post-HCT. The primary endpoints were survival and CR (total serum bilirubin <2 mg/dL and MOF resolution per investigator’s assessment) at Day 100 post-HCT in patients with severe/very severe VOD/SOS. Key secondary endpoints included the evaluation of treatment-emergent serious adverse events (SAEs) of interest: haemorrhage, coagulopathy, infection, hypotension, septicaemia, and thromboembolic event, irrespective of relationship to defibrotide treatment; overall mortality and VOD/SOS-related mortality; prognostic factors with an impact on CR or survival; and the rate of graft-versus-host disease (GvHD) post-HCT. Outcomes were also evaluated in patients with mild/moderate VOD/SOS and patients who received defibrotide for prophylaxis of VOD/SOS.

### Statistical analyses

Statistical analyses were performed using SAS software (v9.4; SAS Institute, Cary, NC). Survival and CR of patients with severe/very severe VOD/SOS at Day 100 were estimated using the Kaplan–Meier (KM) method. For survival at Day 100 post-HCT and CR at Day 100 post-HCT, univariate analyses identified prognostic factors at the 20% significance level or factors of clinical interest, which were then selected for a multivariate analysis using a Cox proportional hazard model. The final model was based on the selection of variables by a stepwise selection method, with a 20% input and a 5% output threshold.

## Results

### Patient demographics and clinical characteristics

**Fig. 1:**
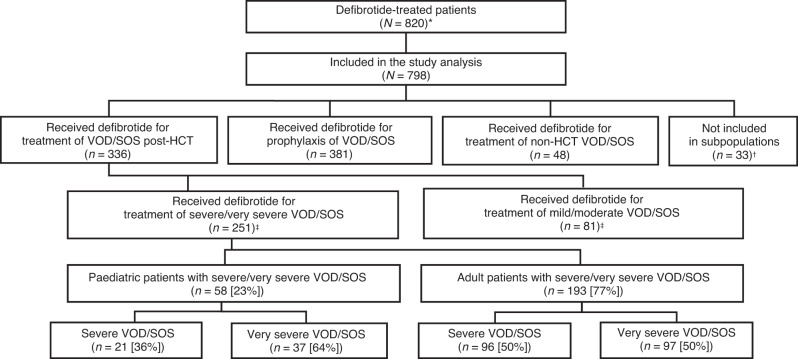
Flow diagram of patients enrolled in DEFIFrance. HCT indicates haematopoietic cell transplantation, VOD/SOS, veno-occlusive disease/sinusoidal obstruction syndrome. *Twenty-two patients in total were excluded from the study analysis: 20 patients received defibrotide prior to the 15 July 2014 start date or were missing a treatment start date; two patients were included in the HARMONY study. ^†^Thirty-three patients were not included in subpopulations: 30 patients had suspected but unconfirmed VOD/SOS; one patient received a liver and kidney transplant rather than HCT; one patient was treated for thrombotic microangiopathy; and one patient received defibrotide for the treatment of VOD/SOS, but data on whether the treatment was given post-HCT were missing. ^‡^Data on the severity of VOD/SOS were missing in four patients.

Overall, 820 patients were enrolled (Fig. 1). Of the 798 patients included in the study analysis, 381 received defibrotide for VOD/SOS prophylaxis and 336 received defibrotide for the treatment of VOD/SOS post-HCT (all severity grades). Data for 48 patients who were treated with defibrotide for non-HCT VOD/SOS and 33 patients who did not meet criteria for inclusion in any specific subpopulation are not reported (Fig. 1).

The primary study population included 251 defibrotide-treated patients with severe/very severe VOD/SOS post-HCT (severe: *n* = 117; very severe: *n* = 134); 81 patients had mild/moderate disease, and severity data were missing in four patients. In the primary study population, the median age was 45 years (range: 0, 74) and 58/251 (23%) patients were <18 years of age (Table 1). The most common primary diagnoses were acute myeloid leukaemia (AML; 68/251 [27%]) and acute lymphoblastic leukaemia (ALL; 49/251 [20%]). Paediatric patients were more likely than adults to have a primary diagnosis of ALL (31% vs. 16%) or neuroblastoma (29% vs. 0%), while AML (12% vs. 32%) and lymphoma (2% vs. 23%) were more common in adults (Supplementary Table 2).

**Table 1:** Baseline demographics and clinical characteristics.

Characteristic	Primary study population: Severe/very severe VOD/SOS post-HCT (*N* = 251)	Mild/moderate VOD/SOS post-HCT (*N* = 81)
Median (range) age at HCT, years	45 (0, 74)	35 (0, 69)
Age group, *n/N* (%)		
<18 years	58/251 (23)	29/81 (36)
≥18 years	193/251 (77)	52/81 (64)
Primary disease,^a^ *n/N* (%)		
AML	68/251 (27)	20/81 (25)
ALL	49/251 (20)	16/81 (20)
Lymphoma	46/251 (18)	10/81 (12)
MDS/MPS	29/251 (12)	12/81 (15)
Conditioning regimen, *n/N* (%)		
Myeloablative	135/250 (54)	61/81 (75)
Allogeneic HCT, *n/N* (%)	220/250 (88)	66/81 (81)
Unrelated donor	99/220 (45)	34/81 (42)
Haploidentical donor	41/220 (19)	8/81 (10)
Prophylaxis for GvHD, *n/N* (%)	218/220 (99)	66/66 (100)
Sirolimus	4/214 (2)	0
Cyclophosphamide post-HCT	48/215 (22)	10/65 (15)
Methotrexate	75/215 (35)	29/65 (45)

*ALL* indicates acute lymphoblastic leukaemia, *AML* acute myeloid leukaemia, *GvHD* graft-versus-host disease, *HCT* haematopoietic cell transplantation, *MDS/MPS* myelodysplastic syndrome/myeloproliferative syndrome, *VOD/SOS* veno-occlusive disease/sinusoidal obstruction syndrome.

^a^Primary disease indicates those occurring in >10% of patients with severe/very severe VOD/SOS post-HCT (primary study population).

The majority (220/250 [88%]) of patients with severe/very severe VOD/SOS had most recently received an allogeneic HCT, 99/220 (45%) of whom had an unrelated donor (Table 1). Allogeneic transplant was less common in paediatric patients (41/58 [71%]) than adults (179/192 [93%]), but the proportion of patients with unrelated donors was comparable (18/41 [44%] vs. 81/179 [45%]; Supplementary Table 2). More than half (135/250 [54%]) of patients received myeloablative conditioning, which was more common in paediatric patients (54/58 [93%]) than in adults (81/192 [42%]; Table 1 and Supplementary Table 2). The most common risk factor for VOD/SOS was prior treatment with hepatotoxic drugs (150/251 [60%]); in addition, prior exposure to gemtuzumab ozogamicin or inotuzumab ozogamicin specifically was a risk factor for VOD/SOS in 23/251 (9%) patients. Other common (>50%) risk factors for VOD/SOS included iron overload (133/231 [58%]), and relapsed/refractory disease (137/251 [55%]; Table 2). Relapsed/refractory disease was less common in paediatric patients (22/58 [38%]) than adults (115/193 [60%]; Supplementary Table 3), and fewer paediatric patients (3/53 [6%]) than adults (26/186 [14%]) had received a second HCT.

**Table 2:** VOD/SOS risk factors.

Characteristic	Primary study population: Severe/very severe VOD/SOS post-HCT (*N* = 251)	Mild/moderate VOD/SOS post-HCT (*N* = 81)
Patient-related risk factors, *n/N* (%)		
Advanced disease (>CR 2 or relapsed/refractory disease)	137/251 (55)	38/81 (47)
Karnofsky or Lansky score <90%	53/240 (22)	13/77 (17)
Second HCT	29/239 (12)	5/61 (8)
Transplant-related risk factors, *n/N* (%)		
Myeloablative conditioning	135/250 (54)	61/81 (75)
Hepatic risk factors,^a^ *n/N* (%)		
Prior treatment with hepatotoxic drugs^b^	150/251 (60)	53/81 (65)
Iron overload (ferritin >1000 ng/mL)	133/231 (58)	30/62 (48)
Transaminases >2.5 ULN	43/250 (17)	9/81 (11)
Bilirubinaemia >1.5 ULN	32/250 (13)	11/81 (14)
Abdominal irradiation or hepatitis	29/251 (12)	11/81 (14)
Prior treatment with GO or IO	23/251 (9)	9/81 (11)

*CR* indicates complete response, *GO* gemtuzumab ozogamicin, *HCT* haematopoietic cell transplantation, *IO* inotuzumab ozogamicin, *ULN* upper limit of normal, *VOD/SOS* veno-occlusive disease/sinusoidal obstruction syndrome.

^a^Risk factors occurring in >5% of patients with severe/very severe VOD/SOS post-HCT (primary study population).

^b^Per the investigators’ discretion; hepatotoxic drugs were not defined in the protocol.

### VOD/SOS diagnosis, grading, and treatment

**Fig. 2:**
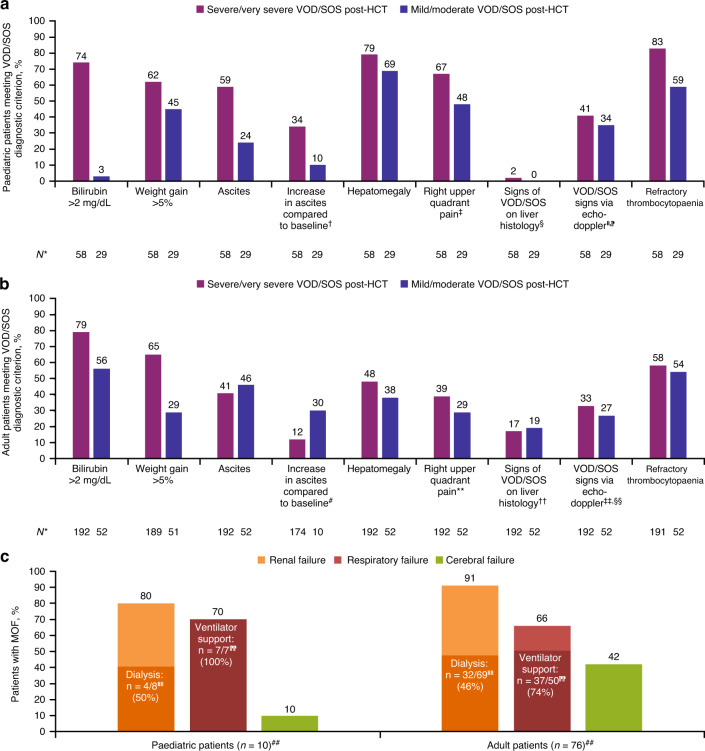
VOD/SOS signs and symptoms and presence of MOF at diagnosis. VOD/SOS signs and symptoms in (**a**) paediatric and (**b**) adult patients. **c** Presence of MOF at diagnosis of VOD/SOS in paediatric and adult patients. HCT indicates haematopoietic cell transplantation, MOF multiorgan failure, VOD/SOS veno-occlusive disease/sinusoidal obstruction syndrome. ^*^Denominator reflects patients with available data on the corresponding criterion. ^†^Data were not determined in 13 patients with severe/very severe VOD/SOS, and in three patients with mild/moderate VOD/SOS. ^‡^Data were not determined in one patient with severe/very severe VOD/SOS, and in one patient with mild/moderate VOD/SOS. ^§^Not applicable in 50 patients with severe/very severe VOD/SOS, and in 28 patients with mild/moderate VOD/SOS who did not have liver histology. ^‖^Not applicable in 15 patients with severe/very severe VOD/SOS, and in 13 patients with mild/moderate VOD/SOS. ^⁋^Abdominal ultrasound was established at baseline in 18 patients with mild/moderate VOD/SOS, and in 43 patients with severe/very severe VOD/SOS. Doppler baselines were established in 8 patients with mild/moderate VOD/SOS, and in 29 patients with severe/very severe VOD/SOS. ^#^Data were not determined in 41 patients with severe/very severe VOD/SOS, and in two patients with mild/moderate VOD/SOS. ^**^Data were not determined in seven patients with severe/very severe VOD/SOS, and in two patients with mild/moderate VOD/SOS. ^††^Not applicable in 127 patients with severe/very severe VOD/SOS, and in 36 patients with mild/moderate VOD/SOS who did not have liver histology. ^‡‡^Not applicable in 44 patients with severe/very severe VOD/SOS, and in 17 patients with mild/moderate VOD/SOS. ^§§^Abdominal ultrasound was established at baseline in six patients with mild/moderate VOD/SOS, and in 128 patients with severe/very severe VOD/SOS. Doppler baselines were established in one patient with mild/moderate VOD/SOS, and in 66 patients with severe/very severe VOD/SOS. ^‖‖^Denominator reflects number of patients with renal failure. ^⁋⁋^Denominator reflects number of patients with respiratory failure. ^##^Denominator reflects number of patients with MOF.

At diagnosis, 55/250 (22%) patients had anicteric VOD/SOS (bilirubin ≤2 mg/dL), which was observed in 15/58 (26%) paediatric and 40/192 (21%) adult patients. Ascites-related symptoms, hepatomegaly, right upper quadrant pain, and refractory thrombocytopaenia were seen more frequently in paediatric than adult patients (Fig. 2a, b). In adults, those meeting the criteria for severe/very severe VOD/SOS most commonly had onset of symptoms within 4 days of diagnosis; the second criterion indicating that VOD/SOS was severe/very severe varied, although bilirubin doubling within 48 h and weight gain ≥5% were frequently present (Supplementary Table 4). In paediatric patients, VOD/SOS was frequently graded as severe/very severe due to a combination of hyperbilirubinaemia with one of the other criteria for severe or very severe disease, such as consistent increase in bilirubin or weight gain over 3 consecutive days. At diagnosis, 86/250 (34%) patients had MOF, which was present in fewer paediatric patients (10/58 [17%]) than adults (76/192 [40%]; Fig. 2c). Regardless of age group, approximately half of patients with renal failure required dialysis and most patients with respiratory failure (77%) required respirator support.

The median (interquartile range [IQR]) time from HCT to VOD/SOS diagnosis was 13 (8, 20) days, and the median (IQR) time from VOD/SOS diagnosis to defibrotide administration was 0 (0, 1) days. In total, 134/247 (54%) patients received 25 mg/kg/day of defibrotide, consistent with the dose recommended in the label; the median (IQR) dose was 25 (25, 25) mg/kg/day. The median (IQR) treatment duration was 18 (11, 22) days in patients with severe and 16 (8, 22) days in those with very severe VOD/SOS post-HCT.

### Resolution of VOD/SOS

**Fig. 3:**
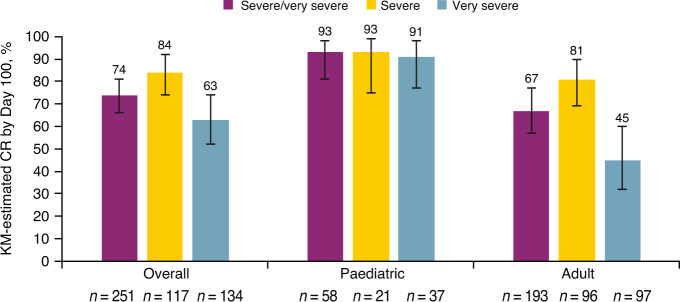
KM-estimated CR by Day 100. CI indicates confidence interval, CR complete response, HCT haematopoietic cell transplantation, KM Kaplan–Meier, VOD/SOS veno-occlusive disease/sinusoidal obstruction syndrome.

At Day 100, 153/251 (61%) patients with severe/very severe VOD/SOS were alive and 137/251 (55%) had CR (49/58 [84%] paediatric patients and 88/193 [46%] adults). The KM-estimated CR by Day 100 post-HCT was 74% (95% confidence interval [CI]: 66%, 81%) in patients with severe/very severe VOD/SOS, with a higher CR at Day 100 observed in patients with severe (84%) than very severe VOD/SOS (63%; Fig. 3). This difference in resolution was only seen for adults (severe, 81%; very severe, 45%); resolution of severe and very severe VOD/SOS was equally high for paediatric patients (severe, 93%; very severe, 91%; Fig. 3).

### Survival post-HCT

**Fig. 4:**
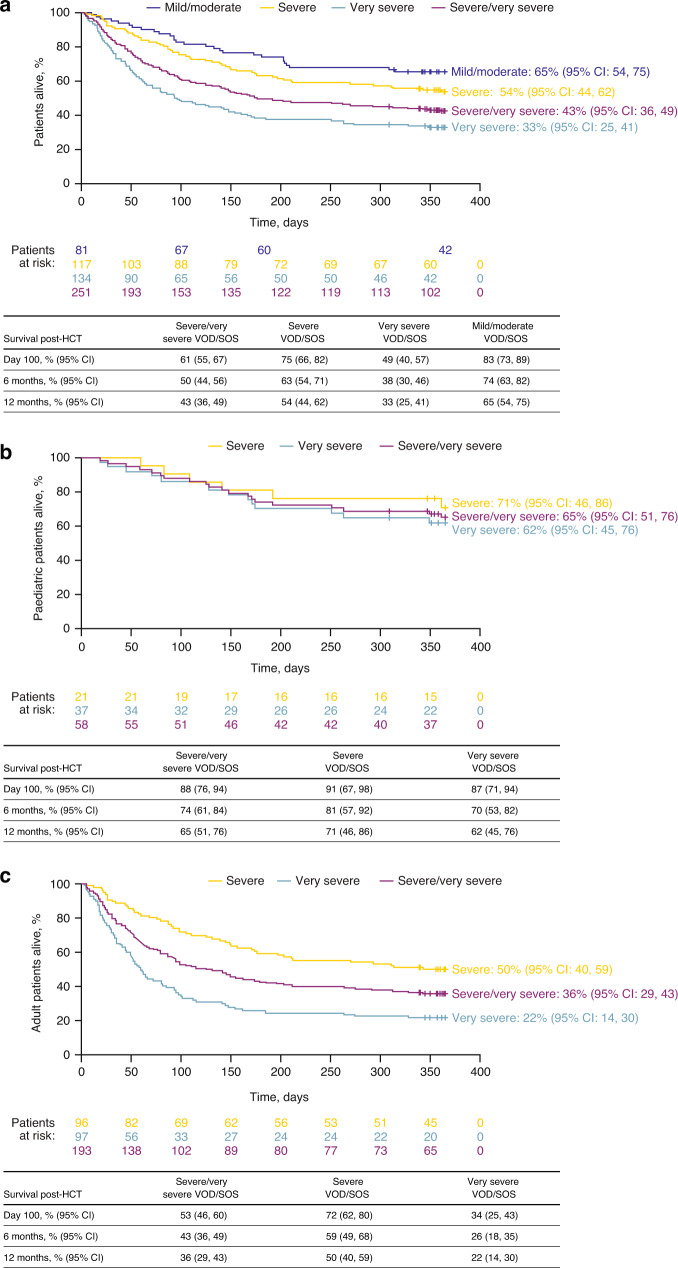
KM-estimated survival. KM-estimated survival (**a**) overall and (**b**) in paediatric and (**c**) adult patients. CI indicates confidence interval, HCT haematopoietic cell transplantation, KM Kaplan–Meier, VOD/SOS veno-occlusive disease/sinusoidal obstruction syndrome.

KM-estimated Day 100 post-HCT survival was 61% (95% CI: 55%, 67%) in patients with severe/very severe VOD/SOS; this was higher for patients with severe (75%) compared to very severe (49%) disease at Day 100, with similar observations at 6 months and 12 months (Fig. 4a). Among paediatric patients, KM-estimated survival by Day 100 was similar between those with severe (91%) and very severe (87%) VOD/SOS (Fig. 4b). Among adults, KM-estimated Day 100 post-HCT survival was higher for those with severe (72%) compared to very severe (34%) VOD/SOS at all time points (Fig. 4c).

### Factors associated with VOD/SOS resolution and survival

By multivariate Cox analysis of CR at Day 100, the chance of achieving CR was evaluated using the hazard ratio (HR). From this analysis, adult patients (HR vs. paediatric patients = 0.55; *P* = 0.004), those who needed dialysis (HR vs. no need for dialysis = 0.36; *P* = 0.028), and patients who displayed cognitive failure (HR vs. the absence of cognitive failure = 0.26; *P* = 0.025) were less likely to achieve CR by Day 100. In a similar analysis of survival, multivariate Cox analysis demonstrated that adults (HR vs. paediatric patients = 3.27; *P* = 0.004), patients who required dialysis (HR vs. no need for dialysis = 2.56; *P* = 0.0001), and those with respiratory failure (HR vs. patients without respiratory failure = 2.73; *P* < 0.0001) were more likely to die by Day 100. However, patients who received myeloablative conditioning (HR vs. patients receiving a reduced-intensity conditioning regimen = 0.59; *P* = 0.019) were less likely to die by Day 100 compared to those who received a reduced-intensity conditioning regimen.

### SAEs of interest

Treatment-emergent SAEs of interest occurred in 29% of patients with severe/very severe VOD/SOS post-HCT (Table 3). The most common (≥5% of patients) treatment-emergent SAE categories were infection (17%) and haemorrhage (16%). Common (≥1% of patients) individual SAEs are shown in Table 3.

**Table 3:** Treatment-emergent SAEs of interest (occurring in ≥1% of patients in the primary study population).

	Post-HCT VOD/SOS	
	Primary study population: Severe/very severe VOD/SOS post-HCT (*N* = 251)	Mild/moderate VOD/SOS post-HCT (*N* = 81)
Any treatment-emergent SAE of interest, *n* (%)	72 (29)	23 (28)
Infection, *n* (%)	43 (17)	6 (7)
Infection NOS	10 (4)	2 (2)
Cytomegalovirus infection	8 (3)	0
BK virus infection	6 (2)	1 (1)
Cytomegalovirus infection reactivation	5 (2)	2 (2)
Aspergillus infection	4 (2)	0
Haemorrhage, *n* (%)	40 (16)	15 (19)
Haemorrhagic cystitis	9 (4)	2 (2)
Haemorrhage NOS	8 (3)	2 (2)
Gastrointestinal haemorrhage	5 (2)	2 (2)
Pulmonary alveolar haemorrhage	4 (2)	0
Viral haemorrhagic cystitis	4 (2)	3 (4)
Hypotension, *n* (%)	6 (2)	1 (1)
Coagulopathy, *n* (%)	3 (1)	2 (2)
Thromboembolism, *n* (%)	2 (1)	2 (2)
Septicaemia, *n* (%)	2 (1)	0

*HCT* indicates haematopoietic cell transplantation, *NOS* not otherwise specified, *SAE* serious adverse event, *VOD/SOS* veno-occlusive disease/sinusoidal obstruction syndrome.

### Mortality

**Fig. 5:**
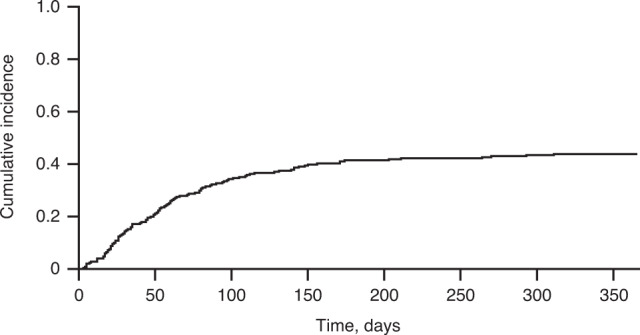
Cumulative incidence of HCT-related mortality in patients with severe/very severe VOD/SOS at 12 months post-HCT. HCT indicates haematopoietic cell transplantation.

Among patients with severe/very severe VOD/SOS, the cumulative incidence of death due to VOD/SOS by 12 months, with death from other causes as a competing event, was 15%. This was 6% for severe and 23% for very severe VOD/SOS. Other common (>25% of HCT-related patient deaths) causes of HCT-related death were infection, MOF, renal toxicity, and GvHD, according to physician evaluation. The cumulative incidence of HCT-related mortality at 12 months is shown in Fig. 5.

### Development of acute GvHD

Among patients who received defibrotide for treatment of severe/very severe VOD/SOS after an allogeneic HCT (*n* = 220), 21/41 (51%) paediatric patients and 62/179 (35%) adults developed acute GvHD grade 2–4 by Day 100 post-transplant.

### Patients receiving defibrotide for treatment of mild/moderate VOD/SOS post-HCT

Among the 81 patients diagnosed with mild/moderate VOD/SOS, the median age was slightly younger than those diagnosed with severe/very severe VOD/SOS (35 vs. 45 years), which was driven by a higher proportion of paediatric patients in the mild/moderate (36%) versus severe/very severe (23%) VOD/SOS groups (Table 1). The proportions of patients with key risk factors were generally similar to those seen for the severe/very severe population (Table 2). Anicteric VOD/SOS was much more frequent in patients with mild/moderate VOD/SOS (63%) compared to those with severe/very severe VOD/SOS (22%). Among patients with mild/moderate disease, CR at Day 100 post-HCT was 68%. KM-estimated survival was numerically higher in patients with mild/moderate VOD/SOS than in those with severe/very severe VOD/SOS at all time points (Fig. 4a). Treatment-emergent SAEs of interest occurred in 28% of patients; the most common categories were haemorrhage and infection (Table 3).

### Patients receiving defibrotide for prophylaxis of VOD/SOS

In total, 381 patients received defibrotide for prophylaxis of VOD/SOS (178 [47%] paediatric patients and 203 [53%] adults). The population represented a high-risk group of patients, with ≥90% having ≥2 factors recognised as increasing the risk of developing VOD/SOS. Notably, 39% had received ≥2 HCTs, and 30% had received prior inotuzumab ozogamicin or gemtuzumab ozogamicin. By Day 30 post-HCT, 76/381 (20%) patients had developed VOD/SOS; of these, 32% had mild/moderate, 34% had severe, and 34% had very severe VOD/SOS. A total of 50/178 (28%) paediatric patients and 26/203 (13%) adults developed VOD/SOS by Day 30 post-HCT. Altogether, 25% of patients receiving defibrotide for prophylaxis of VOD/SOS experienced treatment-emergent SAEs of interest. The most common (≥5%) treatment-emergent SAE categories were haemorrhage (14%) and infection (13%).

## Discussion

The DEFIFrance study represents the largest collection of real-world data on the post-registration use of defibrotide, indicated for the treatment of severe hepatic VOD/SOS post-HCT. Clinical studies have demonstrated favourable efficacy and safety of defibrotide for the treatment of patients with VOD/SOS, and additional data regarding the real-world use of defibrotide are beneficial to further support its use in clinical practice. The effectiveness and safety outcomes observed in this real-world setting study align with prior studies that support the use of defibrotide for the treatment of VOD/SOS post-HCT in adult and paediatric patients.

Given the life-threatening nature of severe VOD/SOS, persistent vigilance for signs and symptoms of VOD/SOS and prompt intervention upon diagnosis are critical. More sensitive criteria have always been needed to allow prompt diagnosis and management of VOD/SOS before patients reach the most severe stages. Attempts have also been made to reduce post-HCT complications, including VOD/SOS, such as the development of alternative donors and reduced-intensity regimens, which have led to changes in HCT practice and associated risk factors [14]. Additional factors complicating the diagnosis of VOD/SOS are the heterogeneous and dynamic manner in which symptoms present, with some patients not exhibiting all of the classical features of the disease. In the DEFIFrance study, classical signs and symptoms used for diagnosis (e.g. hyperbilirubinaemia, ascites, and weight gain) were reported in most patients with severe/very severe VOD/SOS post-HCT. However, more than 20% of these patients had bilirubin ≤2 mg/dL at VOD/SOS diagnosis and would not have been identified by diagnostic criteria requiring bilirubin >2 mg/dL, such as the Baltimore criteria. The occurrence of anicteric VOD/SOS in this study is consistent with an expanded access study, which reported that 23% of patients diagnosed with VOD/SOS were anicteric cases [16]. The diagnostic criteria employed can clearly affect the rates of VOD/SOS observed. For example, in an analysis of 135 studies in patients with VOD/SOS, the incidence of VOD/SOS was 17.3% using the Seattle criteria and 9.6% using the Baltimore criteria [1]. In another retrospective study of 4290 patients receiving allogeneic HCT, the cumulative incidence of VOD/SOS diagnosed using the Seattle criteria was 10.8% (*n* = 462) and using the modified Seattle criteria was 9.3%; only 107 patients met the more stringent Baltimore criteria [17]. Moreover, a single-centre, retrospective study of paediatric patients, adolescents, and young adults who underwent HCT compared rates of VOD/SOS diagnosis using the Baltimore and modified Seattle diagnostic criteria to those determined by the updated paediatric EBMT guidelines. In this study, a higher incidence of VOD/SOS was identified retrospectively using the paediatric EBMT criteria (15.9%) compared to the modified Seattle criteria (12.3%) and Baltimore criteria (6.6%) [18]. This finding suggests that the newer paediatric EBMT criteria are better at detecting subtle cases that may be missed by the Baltimore and Seattle/modified Seattle criteria. These examples illustrate how diagnostic criteria can affect the numbers of patients diagnosed, which may affect patient outcomes. Taken together, these data highlight the need for vigilance for other signs and symptoms of VOD/SOS in the absence of hyperbilirubinaemia and argue for the use of diagnostic criteria that acknowledge VOD/SOS without hyperbilirubinaemia in both paediatric and adult patients.

Consistent with evidence from prior studies of defibrotide treatment in the real-world setting, effectiveness and safety data from DEFIFrance support the utility of defibrotide for the treatment of patients with severe or very severe VOD/SOS post-HCT. In DEFIFrance, KM-estimated Day 100 post-HCT survival was 61% in the primary study population, comprising patients with severe or very severe VOD/SOS. These findings are similar to results from the previous expanded access and compassionate use studies that reported Day 100 survival of 59% and 58%, respectively, in patients with severe VOD/SOS post-HCT who received 25 mg/kg per day of defibrotide [9, 11]. In a post-authorisation safety study performed by the EBMT, the Day 100 post-HCT survival rate was 73% in patients diagnosed with severe VOD/SOS per the investigator’s assessment [10]. In contrast, the prognosis of VOD/SOS post-HCT without the use of defibrotide may not be as promising. According to a systematic literature review, the overall mortality rate from severe VOD/SOS post-HCT was 84% and mortality exceeded 75% when only supportive treatment was available [1]. Furthermore, a phase 3 study of patients with MOF and VOD/SOS post-HCT found that defibrotide treatment resulted in Day 100 post-HCT survival of 38% versus 25% in a historical control comparator group [8]. Moreover, in an exploratory analysis of defibrotide treatment that included only patients with severe VOD/SOS, defined as VOD/SOS with renal and/or pulmonary dysfunction, Day 100 post-HCT survival was 39% in patients who received defibrotide versus 31% in patients who did not receive defibrotide [19]. A number of other agents have been studied for their potential benefit in the treatment of VOD/SOS, such as tissue plasminogen activator and N-acetylcysteine, but these have not demonstrated significant benefit [2, 20, 21].

Among patients receiving defibrotide for the treatment of VOD/SOS post-HCT, survival was better in patients with less severe disease (when comparing severe vs. very severe VOD/SOS), highlighting the importance of prompt VOD/SOS diagnosis and treatment, before patients reach the most severe stage of VOD/SOS. Although it is not certain that all cases of VOD/SOS will progress in severity, timely diagnosis and administration of defibrotide treatment have been associated with improved outcomes [11, 18]. A similar pattern was observed for CR by Day 100.

It is also worth noting that Day 100 survival and CR were higher in the primary analysis population in DEFIFrance (patients with severe/very severe VOD/SOS) compared to the pivotal phase 3 trial of defibrotide in patients with severe VOD/SOS by Richardson et al. [8]. In the current study, patients with less severe VOD/SOS, including anicteric patients, could be enrolled, whereas patients in the Richardson et al. study were required to have VOD/SOS, diagnosed by Baltimore criteria (requiring elevated bilirubin) with MOF post-HCT. Because all patients were required to have MOF, by EBMT severity criteria, all patients in the previous phase 3 trial would have been considered to have very severe VOD/SOS. In contrast, the overall severity of VOD/SOS among patients in the primary analysis population of DEFIFrance (severe/very severe) was, by definition, less severe than that in the prior phase 3 trial, which may explain the higher survival and CR response in the current study. However, whereas survival and CR were higher in adult patients with severe versus very severe VOD/SOS post-HCT, this was not observed among paediatric patients, possibly because survival and CR were relatively high in paediatric patients, making differences between severity groups harder to discern. Another possible explanation is that there are few differences in the paediatric EBMT criteria for classification of severe and very severe VOD/SOS.

The current study also included patients receiving defibrotide for prophylaxis of VOD/SOS, of whom 20% developed VOD/SOS by Day 30 post-HCT. The absence of an untreated control group limits the ability to determine whether defibrotide prophylaxis was successful in reducing VOD/SOS incidence by Day 30 post-HCT in this high-risk group of patients. These patients had multiple risk factors that increased the odds of developing VOD/SOS, thereby necessitating prophylaxis with defibrotide. That said, in a separate meta-analysis of more than 3000 patients, the risk ratio for developing VOD/SOS with defibrotide prophylaxis versus control was 0.30 (95% CI: 0.12, 0.71; nominal *P* = 0.006) [22]. Moreover, results from a phase 3, prospective, randomised controlled study in high-risk paediatric patients demonstrated a 12% incidence of VOD/SOS by Day 30 post-HCT with defibrotide prophylaxis, while an incidence of 20% was observed in the no defibrotide control group [23]. Similarly, in the non-interventional EBMT PASS registry, using the EBMT database, the incidence of VOD/SOS among 76 high-risk patients, who received defibrotide for VOD/SOS prophylaxis, 9 (12%) developed VOD/SOS [24]. It can be speculated that patients in the DEFIFrance study who received defibrotide prophylaxis may have been at a particularly high risk of VOD/SOS than in the prior phase 3 prospective and EBMT PASS studies, and thus had higher incidence of VOD/SOS. Although, direct comparison of results across trials is tenuous. Defibrotide prophylaxis was also recently studied in patients at high risk or very high risk for developing VOD/SOS post-HCT versus best supportive care for the prevention of VOD/SOS in the phase 3, open-label, randomised, adaptive HARMONY study [25]. No significant difference was observed in the defibrotide prophylaxis versus best supportive care groups in the primary endpoint of VOD/SOS-free survival by Day 30 post-HCT (when VOD/SOS was diagnosed by an Endpoint Adjudication Committee). However, VOD/SOS-free survival by Day 30 post-HCT was numerically higher in the defibrotide prophylaxis group compared to the best supportive care group when VOD/SOS was diagnosed by the investigators.

The safety profile of defibrotide for the treatment of VOD/SOS post-HCT was consistent with previous real-world studies [9–11]. Similar to the EBMT PASS conducted in Europe [10], the incidence of SAEs of interest was ~25% to 30% for patients receiving treatment for VOD/SOS post-HCT. The most frequently reported SAEs in patients with severe/very severe VOD/SOS post-HCT in the current study were infection (17%) and haemorrhage (16%), which is also consistent with findings from the EBMT PASS (infection, 24%; bleeding, 13%) [10]. Throughout these studies, incidences of SAEs of special interest were lower in paediatric than adult patients, regardless of disease severity, which may be related, in part, to the better survival and CR outcomes observed in paediatric patients compared to adults.

There are some limitations to this study. As a registry study, DEFIFrance was limited by the data reported on the study form. The lack of a control arm evaluating patients with VOD/SOS post-HCT who were not treated with defibrotide limits the interpretation of the data. Although it is likely that all patients with severe VOD/SOS would receive defibrotide based on its indication, data from any patients who developed VOD/SOS post-HCT and did not receive defibrotide were not collected, which may have provided an insightful comparison of its overall benefit. In addition, there was no requirement for the use of a single set of VOD/SOS diagnostic criteria because the diagnostic criteria used was dependent on physician practice and institution protocols. Thus, the utilisation of various diagnostic criteria may be a potential source of heterogeneity in the DEFIFrance study.

In conclusion, the DEFIFrance study represents the largest collection of real-world data on the post-registration use of defibrotide. Among patients receiving defibrotide for VOD/SOS post-HCT, outcomes were better in patients with severe versus very severe VOD/SOS, which highlights the importance of early VOD/SOS diagnosis and treatment before patients reach the most severe stage of VOD/SOS. The effectiveness and safety observed in this real-world setting study add to evidence from prior studies supporting the utility of defibrotide for treating paediatric and adult patients with severe/very severe VOD/SOS post-HCT.

## Supplementary information

Supplementary Information

## Data Availability

All relevant data are provided within the paper and supplemental files. Jazz has established a process to review requests from qualified external researchers for data from Jazz-sponsored clinical trials in a responsible manner that includes protecting patient privacy, assurance of data security and integrity, and furthering scientific and medical innovation. Additional details on Jazz Pharmaceuticals data-sharing criteria and process for requesting access can be found at: https://www.jazzpharma.com/science/clinical-trial-data-sharing/. All authors had access to the clinical trial data relevant for the publication.
