# Supplementary material for: Real-world use of defibrotide for veno-occlusive disease/sinusoidal obstruction syndrome: the DEFIFrance Registry Study
Source: Bone Marrow Transplant. 2022 Dec 23;58(4):367–76. doi: 10.1038/s41409-022-01900-6 (PMC10073014; doi:10.1038/s41409-022-01900-6)
Supplement: Supplementary file 1 — Supplementary Information [file 41409_2022_1900_MOESM1_ESM.pdf]

Supplementary Table 1. List of Investigators

**Principal investigator**	**Name of centre, city**
Jacques Olivier Bay	CHU Estaing, Clermont-Ferrand
Ana Berceanu	CHU Besançon, Hôpital Jean-Minjoz, Besançon
Karin Bilger	ICANS-Institut de Cancérologie Strasbourg, Strasbourg
Laurence Blanc	CHU Poitiers, Hôpital La Milétrie, Poitiers
Damien Bodet	CHU Caen, Hôpital Côte de Nacre, Caen
Jean Henri Bourhis	Département d'Hématologie, Institut Gustave Roussy, Villejuif
Benedicte Bruno	CHU de Lille, Hôpital Jeanne de Flandre, Lille
Sylvain Chantepie	CHU de Caen Normandie, Hôpital Côte de Nacre, Caen
Pascal Chastagner	CHU Nancy, Hôpital de Brabois, Nancy
Laurence Clement	CHU Bordeaux, Hôpital Haut-Lévêque, Pessac
Jerome Cornillon	CHU Nord St Étienne, Saint-Priest-en-Jarez
Marie-Anne Couturier	CHRU Brest, Hôpital Morvan, Brest
Jean-Hughes Dalle	Hôpital Robert-Debré, GHU APHP Nord et Université de Paris, Paris
Christelle Dufour	Institut Gustave Roussy, Villejuif

**Principal investigator**	**Name of centre, city**
Nathalie Fegueux	CHRU Montpellier, Hôpital St Eloi, Montpellier
Marion Gambart	CHU Toulouse, Hôpital des Enfants, Toulouse
Virginie Gandemer	University Hospital of Rennes, Rennes
Stéphane Girault	CHU Limoges, Hôpital Dupuytren, Limoges
Anne Huynh	Département d'Hématologie, Institut Universitaire du Cancer de Toulouse – Oncopole, Toulouse
Fazeh Izadifar-Legrand	Institut Paoli-Calmettes, Aix Marseille Université, Management Sport Cancer Laboratoire (MSC), Marseille
Charlotte Jubert	CHU Bordeaux, Hôpital des Enfants, Bordeaux
	CHU Estaing, Clermont-Ferrand
Marion Klemencie	CHU Angers, Angers
Hélène Labussiere-Wallet	Hôpital Lyon Sud, Pierre Bénite
Thierry Lamy de la Chapelle	CHU Rennes, Hôpital Pontchaillou, Rennes
Delphine Lebon	CHU Amiens-Picardie, Amiens

**Principal investigator**	**Name of centre, city**
Lauriane Lemelle	SIREDO Oncology Centre (Care, Innovation and Research for Children and AYA with Cancer), Institut Curie, Paris
Natacha Maillard	CHU Poitiers, Hôpital La Milétrie, Poitiers
Jean-Valere Malfuson	Hôpital d'instruction des armées Percy, Clamart
Ambroise Marcais	Hôpital Necker-Enfants maladies, Paris
Sébastien Maury	Hôpital Henri Mondor, Université Paris-Est Créteil Val de Marne (UPEC), Créteil
Anne-Lise Menard	Centre Henri Becquerel, Rouen
Gérard Michel	CHU Marseille, Hôpital Timone, Marseille
Mohamad Mohty	Hôpital St Antoine, Université Sorbonne, INSERM UMRs 938, Paris
Stephanie Nguyen Quoc	Hôpital Pitié-Salpêtrière, Paris
Catherine Paillard	CHU de Hautepierre, Département d'Pédiatrie Hématologie Oncologie, Strasbourg
Régis Peffault de Latour	Hôpital Saint-Louis, Université Paris Cité, Paris
Isabelle Pellier	Univ Angers-CHU Angers- INSERM 1232, CRCINA- F -49000 Angers- France
Arnaud Petit	APHP Hôpital Armand-Trousseau, Paris
Dominique Plantaz	CHU Grenoble Alpes, Pôle Couple Enfant, Grenoble

Principal investigator	Name of centre, city
Aurelie Plessier	Université de Paris, AP-HP, Hôpital Beaujon, Service d'Hépatologie, DMU DIGEST, Centre de Référence des Maladies Vasculaires du Foie, FILFOIE, ERN RARE-LIVER, Centre de recherche sur l'inflammation, Inserm, UMR 1149, Paris
Yves Réguerre	CHR Félix-Guyon, Réunion
Cecile Renard	Institut d’Hématologie et d’Oncologie Pédiatrique, Lyon
Fanny Rialland	CHU de Nantes, Hôpital Femme-Enfant-Adolescent, Nantes
Pierre Simon Rohrlich	CHU Nice, Hôpital L’Archet, Nice
Marie Thérèse Rubio	CHRU Nancy, Hôpitaux de Brabois, Nancy
Celia Salanoubat	Centre Hospitalier Sud Francilien, Corbeil-Essones
Pascale Schneider	CHU de Rouen, Département d’Hémato-oncologie Pédiatrique, Rouen
Anne Sirvent	CHU de Montpellier, Hôpital A De Villeneuve, Montpellier
Carole Soussain	Institut Curie, site de Saint-Cloud
Jean Louis Stephan	CHU Nord St Étienne, Saint-Priest-en-Jarez
Anne Thiebaut	CHU Grenoble Alpes, Hôpital Michallon, La Tronche
Ibrahim Yakoub-Agha	CHU de Lille, Université de Lille, INSERM U1286, Infinite, Lille

Supplementary Table 2. Demographics and Clinical Characteristics by Age Group

	**Primary study population: severe/very severe VOD/SOS post-HCT**		**Mild/moderate VOD/SOS post-HCT**	
	**Paediatric (N = 58)**	**Adult (N = 193)**	**Paediatric (N = 29)**	**Adult (N = 52)**
**Median (range) age at HCT, years**	6 (0, 18)	52 (18, 74)	4 (0, 16)	54 (19, 69)
**Primary disease,* n/N (%)**				
ALL	18/58 (31)	31/193 (16)	6/29 (21)	10/52 (19)
AML	7/58 (12)	61/193 (32)	2/29 (7)	18/52 (35)
Lymphoma	1/58 (2)	45/193 (23)	1/29 (3)	9/52 (17)
MDS/MPS	1/58 (2)	28/193 (15)	2/29 (7)	10/52 (19)
Neuroblastoma/solid tumour	17/58 (29)	0	8/29 (28)	1/52 (2)
Genetic disease or immune deficiency	7/58 (12)	0	4/29 (14)	0
**Conditioning regimen, n/N (%)**				
Myeloablative	54/58 (93)	81/192 (42)	29/29 (100)	32/52 (62)
**Allogeneic HCT, n/N (%)**	41/58 (71)	179/192 (93)	21/29 (72)	45/52 (87)

Unrelated donor	18/41 (44)	81/179 (45)	13/29 (45)	21/52 (40)
Haploidentical donor	2/41 (5)	39/179 (22)	0	8/52 (15)
**Prophylaxis for GvHD, n/N (%)**	41/41 (100)	177/179 (99)	21/21 (100)	45/45 (100)
Sirolimus	1/40 (3)	3/174 (2)	0	0
Cyclophosphamide post-HCT	2/40 (5)	46/175 (26)	0	10/44 (23)
Methotrexate	9/40 (23)	66/175 (38)	8/21 (38)	21/44 (48)

ALL indicates acute lymphoblastic leukaemia; AML, acute myeloid leukaemia; GvHD, graft-versus-host disease; HCT, haematopoietic cell transplantation; MDS/MPS, myelodysplastic syndrome/myeloproliferative syndrome; and VOD/SOS, veno-occlusive disease/sinusoidal obstruction syndrome.

*Primary disease indicates those occurring in >10% of paediatric or adult patients with severe/very severe VOD/SOS post-HCT (primary study population).

Supplementary Table 3. VOD/SOS Risk Factors by Age Group

	Primary study population: Severe/very severe VOD/SOS post-HCT		Mild/moderate VOD/SOS post-HCT	
	**Paediatric (N = 58)**	**Adult (N = 193)**	**Paediatric (N = 29)**	**Adult (N = 52)**
**Patient-related factors,* n/N (%)**				
Advanced disease (>CR 2 or relapsed/refractory disease)	22/58 (38)	115/193 (60)	9/29 (31)	29/52 (56)
Karnofsky or Lansky score <90%	13/53 (25)	40/187 (21)	2/27 (7)	11/50 (22)
Second HCT	3/53 (6)	26/186 (14)	0	5/33 (15)
**Transplant-related risk factors, n/N (%)**				
Myeloablative conditioning	54/58 (93)	81/192 (42)	29/29 (100)	32/52 (62)
**Hepatic risk factors,* n/N (%)**				
Prior treatment with hepatotoxic drugs[†]	26/58 (45)	124/193 (64)	18/29 (62)	35/52 (67)
Iron overload (ferritin >1 000 ng/mL)	29/53 (55)	104/178 (58)	12/24 (50)	18/38 (47)
Transaminase >2.5 ULN	13/58 (22)	30/192 (16)	4/29 (14)	5/52 (10)
Bilirubinaemia >1.5 ULN	6/58 (10)	26/192 (14)	4/29 (14)	7/52 (13)

Abdominal irradiation or hepatitis	6/58 (10)	23/193 (12)	2/29 (7)	9/52 (17)
Prior treatment with GO or IO	4/58 (7)	19/193 (10)	2/29 (7)	7/52 (13)

CR indicates complete remission; GO, gemtuzumab ozogamicin; HCT, haematopoietic cell transplantation; IO, inotuzumab ozogamicin; ULN, upper limit of normal; and VOD/SOS, veno-occlusive disease/sinusoidal obstruction syndrome.

*Risk factors in >5% of patients with severe/very severe VOD/SOS post-HCT (primary study population).

†Per the investigators' discretion; hepatotoxic drugs were not defined in the protocol.

Supplementary Table 4. Criteria for Severity Grading of VOD/SOS

	Primary study population: severe/very severe VOD/SOS post-HCT	
	**Paediatric patients (N = 58)**	**Adult patients (N = 193)**
**Kinetics of onset,[*] n/N (%)**		
>7 days	8/58 (14)	31/192 (16)
5-7 days	12/58 (21)	29/192 (15)
≤4 days	38/58 (66)	132/192 (69)
**Bilirubin, n/N (%)**		
<2 mg/dL	17/58 (29)	43/193 (22)
≥2 mg/dL and <3 mg/dL	16/58 (28)	36/193 (19)
≥3 mg/dL and <5 mg/dL	11/58 (19)	48/193 (25)
≥5 mg/dL and <8 mg/dL	6/58 (10)	34/193 (18)
≥8 mg/dL	8/58 (14)	32/193 (17)
Doubled within 48 h	12/36 (33)	78/191 (41)
**Transaminase, n/N (%)**		

≤2 N	31/58 (53)	102/193 (53)
>2 N and ≤5 N	6/58 (10)	44/193 (23)
>5 N and ≤8 N	6/58 (10)	14/193 (7)
>8 N	15/58 (26)	33/193 (17)
**Weight gain, n/N (%)**		
<5%	23/58 (40)	67/190 (35)
≥5% and <10%	19/58 (33)	70/190 (37)
≥10%	16/58 (28)	53/190 (28)
**Creatinine, n/N (%)**		
<1.2 × level at HCT	22/39 (56)	84/193 (44)
≥1.2 and <1.5 × level at HCT	7/39 (18)	29/193 (15)
≥1.5 and <2.0 × level at HCT	0	26/193 (13)
≥2.0 × level at HCT	10/39 (26)	54/193 (28)
**Criteria exclusive to paediatric patients**		
**Increase in bilirubin from baseline for 3 consecutive days or bilirubin ≥2 mg/dL, n/N (%)**	52/58 (90)	NA

**Weight gain for 3 consecutive days, n/N (%)**	30/49 (61)	NA
**Paediatric glomerular filtration rate, n/N (%)**		NA
89-60 mL/min	27/36 (75)	
59-30 mL/min	6/36 (17)	
29-15 mL/min	3/36 (8)	
<15 mL/min	0	
**Supplemental oxygen required, n/N (%)**	27/58 (47)	NA
**Supportive ventilation required, n/N (%)**	6/16 (38)	NA
**Presence of cognitive impairment, n/N (%)**	4/58 (7)	NA
**Bleeding disorder requiring administration of coagulation factors, n/N (%)**	8/58 (14)	NA

HCT indicates haematopoietic cell transplantation; NA, not applicable; and VOD/SOS, veno-occlusive disease/sinusoidal obstruction syndrome.

*Duration between the date on which the first signs/symptoms of VOD appeared (retrospectively determined) and the date on which the symptoms fully met the diagnosis criteria.
